# A systematic review of surface electromyography analyses of the bench press movement task

**DOI:** 10.1371/journal.pone.0171632

**Published:** 2017-02-07

**Authors:** Petr Stastny, Artur Gołaś, Dusan Blazek, Adam Maszczyk, Michał Wilk, Przemysław Pietraszewski, Miroslav Petr, Petr Uhlir, Adam Zając

**Affiliations:** 1 Department of Sport Games, Faculty of Physical Education and Sport, Charles University, Prague, Czech Republic; 2 Department of Sports Theory, The Jerzy Kukuczka Academy of Physical Education, Katowice, Poland; 3 Faculty of Physical Culture, Palacky University in Olomouc, Olomouc, Czech Republic; University of California, Davis, UNITED STATES

## Abstract

**Background:**

The bench press exercise (BP) plays an important role in recreational and professional training, in which muscle activity is an important multifactorial phenomenon. The objective of this paper is to systematically review electromyography (EMG) studies performed on the barbell BP exercise to answer the following research questions: Which muscles show the greatest activity during the flat BP? Which changes in muscle activity are related to specific conditions under which the BP movement is performed?

**Strategy:**

PubMed, Scopus, Web of Science and Cochrane Central Register of Controlled Trials (CENTRAL) in the Cochrane Library were searched through June 10, 2016. A combination of the following search terms was used: bench press, chest press, board press, test, measure, assessment, dynamometer, kinematics and biomechanics. Only original, full-text articles were considered.

**Results:**

The search process resulted in 14 relevant studies that were included in the discussion. The triceps brachii (TB) and pectoralis major (PM) muscles were found to have similar activity during the BP, which was significantly higher than the activity of the anterior deltoid. During the BP movement, muscle activity changes with exercise intensity, velocity of movement, fatigue, mental focus, movement phase and stability conditions, such as bar vibration or unstable surfaces. Under these circumstances, TB is the most common object of activity change.

**Conclusions:**

PM and TB EMG activity is more dominant and shows greater EMG amplitude than anterior deltoid during the BP. There are six factors that can influence muscle activity during the BP; however, the most important factor is exercise intensity, which interacts with all other factors. The research on muscle activity in the BP has several unresolved areas, such as clearly and strongly defined guidelines to perform EMG measurements (e.g., how to elaborate with surface EMG limits) or guidelines for the use of exact muscle models.

## Introduction

The bench press exercise (BP) plays an important role in recreational and professional training, including power lifting, in which this exercise is a competitive event. The BP is a complex exercise of the upper body, in which great external loads can be lifted, requiring high neuromuscular activity. The potential of this exercise for strength development and BP competitions has created a unique phenomenon of BP as a popular exercise for training, testing and research purposes.

Scientists and coaches are interested in details related to maximum strength, explosive strength improvement or power output during the BP exercise as well as muscle activity between BP variations. For this purpose, two previous reviews have been written. The first review evaluated the criteria for BP efficiency and safety that can be prescribed in conditioning programs [1], and the second evaluated the optimal load for power training [2]. Nevertheless, limited information is available regarding the relationship between BP exercise variations and muscle activity. Regarding previous BP reviews, muscle activity during the BP can help determine which BP variations are effective for increasing the athlete’s performance or neuromuscular adaptation and which variation can strengthen specific muscles during therapy or reconditioning.

The BP may be performed with different grip widths, different speeds of movement and ranges of motion. Exercise intensity is defined by the percentage of 1 repetition maximum (1RM). It seems obvious that all of these variables will affect muscle activation during the BP. Because there is a large variability in performing the BP, appropriate methods must be chosen to describe muscle activity during the BP. When determining EMG data collection methodologies, researchers should follow the most up-to-date recommendations [3, 4]. Therefore, the objective of this paper is to review electromyography (EMG) and kinematic studies performed on resistance trained (RT) subjects in studies with at least one type of cross sectional data collection during the barbell BP exercise. The authors created several principal research questions before beginning this systematic review: Which muscles show the greatest activity during the BP? Which changes in muscle activity are related to specific conditions under which the BP is performed? The results summarize the current knowledge and identify future directions for EMG research on the BP.

## Materials and methods

### Review process

**Fig 1:**
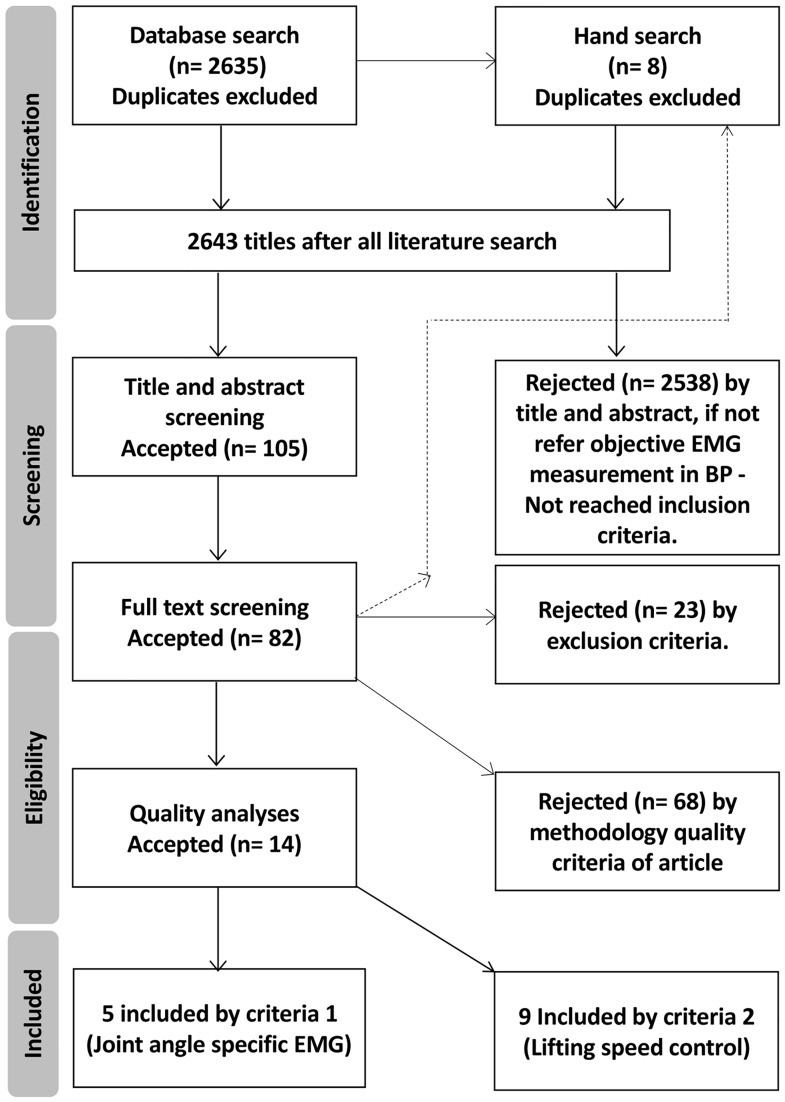
Review flow chart for articles included in tables.

This study utilized the Preferred Reporting Items for Systematic Reviews and Meta Analyses (PRISMA) [5] guidelines during the search and reporting phases. After identifying potential articles, literature screening and full-text selection for eligibility assessment were performed (Fig 1). Eligibility was assessed using the “Strengthening the Reporting of Observational Studies in Epidemiology” (STROBE) checklist [6], which is designed to assess the potential for bias in the study and to assess its generalizability (S1 Table). The STROBE checklist (S2 Table), review protocol (S1 Protocol) and flow diagram (Fig 1) were created and used during the systematic review. The review protocol with data extraction form was stored in the Charles University institutional library (S1 Protocol).

### Literature search

To find articles related to EMG activity during the bench press exercise, a systematic computerized literature search was conducted on June 10, 2016, in PubMed (1940 to search date), Scopus (1823 to search date), Web of Science (1974 to search date) and the Cochrane Central Register of Controlled Trials (CENTRAL) in the Cochrane Library. A combination of the following search terms was used: (bench press) OR (chest press) OR (board press) AND (test OR measure* OR assessment OR dynamometer OR kinematics OR biomechanics). Only original, full-text articles were qualified NOT (Comment [pt] OR Proceeding OR Editorial [pt] OR Letter [pt]). The search did not include conference abstracts and dissertations. Reviews were included to allow a manual search of their reference lists. A manual search of the reference lists of included articles was also performed. The results of the searches were compiled, and duplicates were filtered out (Fig 1).

### Literature selection

After identifying potential articles, the titles and abstracts were reviewed by three independent reviewers (PS, AG, DB) to select relevant articles for full-text screening. The title and abstract screening was focused on three inclusion criteria. First, the studies should include objective electromyography measurements during the bench press (not just BP performance itself). Second, the studied subjects should have RT experience. Third, the study should use a cohort, case control, intervention or cross sectional design. For the first inclusion criterion, the reviewers were asked to answer whether the outcome of the study reported EMG activity in standardized units such as the use of root mean square (RMS) method, mean or peak EMG frequency, mean or peak amplitude or normalized EMG amplitude. For the second criterion, we asked whether the study was performed on subjects with appropriate RT experience. The studies considering BP as a movement pattern require RT subjects to exclude the possible influence of learning or inadequate lifting technique. The third criterion asked whether the study design is a cohort, case control, intervention or a cross sectional study with at least one type of cross sectional data collection. Research questions do not require the use of an intervention to determine the desired outcomes, but the study had to report measurement data under well-controlled conditions. Studies that were identified as a literature or systematic review were designated for a manual search of the reference list. For disagreements regarding the inclusion of articles, agreement was reached by discussion among the reviewers. Full texts of relevant articles were analyzed for final inclusion and eligibility assessment.

Full-text screening was performed by three independent reviewers (PS, MW, PU), who also completed the data extraction form (see Supporting information S1 Protocol). During the full-text screening, the following exclusion criteria were used: 1) the full text was not available in English; 2) the study did not contain an appropriate description of measuring devices, exercise technique or procedures; 3) the study did not include a specific exercise task; and 4) the study did not report how the raw EMG data were processed. In addition, full-text screening included general and specific methodological quality analyses that are described below as well as data collection.

Data collection was performed in studies focused on RT subjects, with a minimum training experience of 6 months. The RT experience also included sport activities in which RT is a common component of conditioning. Study participants had to be 18 years of age and older. Because of the high specificity of study participants, a control group was not required. The EMG signal can be expressed in many different ways; therefore, the reviewers extracted data that were normalized, where the procedure allows data comparison among different studies to answer the first research question. The use of standardized methods such as RMS and frequency analyses allows the determination of differences between particular exercise conditions to answer the second research question. However, these data are not appropriate for comparisons among different studies; therefore, the reviewers extracted only the study results derived from data that were processed by standard procedures.

### Methodological quality

The general methodological estimation of quality was performed using the modified STROBE checklist as part of eligibility assessment; 20 items (S1 Table) evaluated the presence or absence of a quality marker. The absence of a study design (STROBE items 2, 3, 4 in the S1 Table), participant information (STROBE item 6 in the S1 Table), description of the measurement (STROBE item 7 in the S1 Table), reproducibility of the measurement (STROBE item 7 in the S1 Table) and data acquisition (STROBE item 11 in the S1 Table) were used as rejection criteria. Other STROBE items only had an informative value.

The specific methodological quality analyses were composed of two possible criteria to ensure that the EMG data had been collected under well-controlled conditions. The first criterion was the joint angle specificity of gathered EMG because the surface EMG can vary due to electrode shifts toward the innervation zone during dynamic movement [7]. Therefore, EMG studies during isometric contractions or at pre-defined joint angles were included. The second criterion was the pre-defined speed control of measured movements [8]. Therefore, studies that ensured the speed of movement by using isokinetic actions or metronome-based movements were included. This specific eligibility criteria were chosen according to EMG limitations that are described further in the discussion section.

## Results

The database search resulted in 3847 total citations and 2635 articles after removing duplicates. The title and abstract screening resulted in 105 relevant articles that included EMG measurements during the BP task (Fig 1). Of these studies, 23 were rejected following full-text screening, and 68 were rejected based on the methodological quality criteria. Finally, 14 studies (Table 1) were included in the discussion and main tables.

**Table 1:** Basic characteristic of included studies.

Authors	Participants n, gender, age (y), height (cm), BM (kg); specificity	Objective	Muscle model	Movement control type
Clark et. al. 2011 [9]	22, male, 22.7 ± 2.4, 181 ± 7, 94.6 ± 14.5; semi-professional rugby league players	To determine the effect of a 5-week variable ROM training intervention on performance and neuromuscular activation throughout the ROM in well-trained athletes.	PM S, TB long head	AS: 25% of ROM for 3 s
Keogh et. al. 1999 [10]	12, male, 21–35, 177 ± 4.6, 91 ± 14.5; RT performing rugby, powerlifting, bodybuilding, 2–5 RT sessions per week	To gain a stress profile of 7 alternative RT techniques, including isokinetics, eccentrics, functional isometrics, super slow motion, rest pause, breakdowns, and maximal power training, and to compare these RT techniques to heavy weight training.	PM S, TB long head	SC: ecc for 4 s, Isokinetic 0.2 m/s, Super slow 5 s ecc/con AS: Functional isometry for 2 s at 160° of elbow flexion
Snyder and Fry 2012 [11]	11, male, NR, NR, NR; Division III football players, min 6 months of continuous BP experience.	To observe the EMG activity of the agonist and antagonist muscles of RT individuals during a BP exercise at 50% 1RM and 80% 1RM, before and after verbal instructions to subjects to alter the involvement of specified agonist muscles.	PM S, TB long head, AD, Posterior Deltoid, Biceps brachii long head	SC: 2 s ecc/2 s con
Martorelli et. al. 2014 [12]	15, male, 23 ± 3.9, 177 ± 6, 76 ± 7.6; RT: lifting own BM at min.	To examine the effects of upper body graduated compression sleeves on neuromuscular and metabolic responses during a power training.	PM S, TB, AD	SC:, 1.5 s ecc/1.5 s con Smith machine
Moras et. al. 2010 [13]	16, male, 24 ± 3, 180 ± 6.5, 78 ± 8.2; RT experienced	To assess the acute effects on EMG activity during vibration while subjects held the bar in extended and flexed isometric positions during the BP exercise.	PM, TB long head, AD	AS: 180° and 90° elbow flexion for 10–20 s
Rocha et. al. 2007 [14]	13, male, 26 ± 2.5, 175 ± 5.1, 75 ± 8.5; RT lifting own BM at min.	To compare the EMG activity of the PM, AD and TB muscles during the BP and machine peck deck.	PM, TB, AD	SC: 2 s ecc/2 s con
Sakamoto and Sinclair 2012 [15]	13, male, 22 ± 3.6, NR, NR; RT for 3.9 ± 3.2 years, trained 3.2 ± 0.7 sessions/week	To investigated muscle activations under varying speeds and intensities during BP using surface EMG.	PM, TB medial head, AD	SC: at speeds of 5.6, 2.8, 1.9 s per repetition Smith machine
Campos (2014) [16]	12, male, 25 ± 4.3, 176 ± 0.1, 73 ± 6.1; RT 3.6 ± 2.9 y. and min 1 y.	To compare the EMG activity of the selected muscles during dynamic contractions between flat horizontal bench press and barbell pullover exercises.	PM S, PM C, TB long head, AD, Posterior Deltoid, Latissimus dorsi	SC: 2 s ecc/2 s con
Ojasto and Hakkinen (2009) [17]	11, male, 32 ± 4, 178 ± 6.9, NR; Able to lift a 1.2–1.4 times own BM in the BP.	To detect the changes in force responses, muscle activation, and in serum grow hormone and blood lactate in trying to optimize the ecc/con loading protocol used for muscle hypertrophic purposes.	PM, TB, AD, Biceps brachii	AS: during dynamic movement at 90° elbow flexion
Van der Tillaar et. al 2012 [18]	12, male, 22 ± 1.3, 181 ± 5, 78 ± 5.8; RT sport science students	To compare the kinematics and muscle activation patterns of the regular free-weight BP to several isometric BP performed at different distances from the chest.	PM, TB, AD, Biceps brachii	AS: For 3 s at 12 points 3 cm apart during concentric
Norwood et. al. 2007 [19]	10/5, male/female, 29 ± 6.4, 178 ± 8.5, 80 ± 16.5; Elite conditioning coaches RT experience 8.4 y. in average	To examine differences in EMG muscle activation of the trunk stabilizers while subjects were performing BP in stable, single, and dual instability environments.	latissimus dorsi, m.internal obliques, erector spinea, BF, RA. m. soleus,	SC: 2 s ecc/1 s isometric/2 s con
Calatayud et. al 2015 [20]	22/8, men/women 22 ± 2.4, 173 ± 7.6, 71 ± 8.9; RT 2.1 ± 2.4 y. and min 1 y.	To evaluate the EMG levels during 6 RM.	PM S, AD	SC: 2 s ecc/2 s con Smith machine
Calatayud et. al 2016 [21]	18, male, 31 ± 8, 179 ± 8, 82 ± 10; RT 8 ± 6 y., 3RT per week	To evaluate whether focusing on using the PM and TB, respectively, during BP can selectively increase activity of these muscles.	PM S, PM C, TB medial, lateral and long head	SC: 2 s ecc/2 s con
Schoenfeld et. al 2016 [22]	12, male, 22.2 ± 2, 176 ± 6.6, 77 ± 7.1; RT experience 2.8 y.	To compare activation of the upper body musculature during the BP at varying training intensities.	PM S, PM C, TB, AD	SC: 1 s ecc/1 s con

Reported values are the mean ± standard deviation, BM = body mass, ROM = range of motion, EMG = electromyography, RT = resistance training, BF = biceps femoris, RA = rectus abdominis, AS = angle specific, SC = speed control, con = concentric, ecc = eccentric, RM = repetition maximum, min = minimum, PM = pectoralis major, TB = triceps brachii, NR = not reported, AD = anterior deltoid, BP = bench press, S = sternal portion of PM, C = clavicular portion of PM.

Some studies were rejected because the exercise that was reported as a BP was actually performed in a sitting position. The BP exercise is explicitly described as the barbell press from a prone position, where it is possible to recognize the standard flat BP and the competitive powerlifting BP. The standard flat BP requires the exercising subject to have his back in contact with the bench throughout the movement, whereas in powerlifting BP, the athletes are allowed to arch their back. However, no data with the use of EMG from powerlifting BP competition have been reported. However, several studies were excluded because the reported exercise protocol did not match the basic definition of flat BP. Specifically, Wattanaprakornkul [23] and Cacchio [24] reported data from a seated chest press machine, and Patterson [25] reported data from the dumbbell chest press, so these studies were excluded. If the BP was performed on a Smith machine [12] with the appropriate body position according to the authors, then the study was included in the review. However, the AD activity was not included in the results of Smith BP EMG. According to previous findings that barbell BP and Smith machine BP differs in deltoid muscle activity [26] but not TB and PM activity [26, 27], which were included. Two studies were rejected for failing to meet the general methodology description for EMG measurements [28, 29] and 66 by specific methodology criteria 1 and 2.

A total of six selected studies [9–11, 20–22] reported the values of normalized EMG amplitude during BP (Table 2), which can be used to answer our first research question (Which muscles show the greatest activity during the BP?). Two studies [13, 15] based their conclusions on normalized EMG but reported the EMG values only in a graphic form; they were included in the review because it was possible to compare them to other studies. The normalized EMG amplitude was similar for PM and TB [9, 10, 21] and higher for PM than for AD [11, 13, 22]. Moreover, TB can be considered the most sensitive in activity change because TB activity was greater than or less than both the PM and AD in different studies, which indicated that there is no consistent pattern and that other factors play a role. TB activity was lower than PM [11, 22] under isometric conditions [10], lower in the 80% of 1RM condition compared to AD [22] and higher than PM and AD [13] during bar vibration. Based on data that indicate that TB is similar to PM and that PM activity is greater than AD, it can be concluded that PM and TB show greater activity than AD. Therefore, TB and PM have similar activity during the BP, which is significantly higher than the activity of AD.

**Table 2:** Summary of studies that reported normalized muscle electromyography.

Study	Intensity during BP; Normalized muscle activity; EMG units	Main result of study in EMG activity
Clark et. al. 2011 [9]	Max voluntary contraction 3 s isometry at 25% of movement; PM: 131± 30 group one, 141 ± 63 group two, TB: 140 ± 45 group one, 170± 72 group two; %MVIC mean	Training intervention did not have effect on muscle activity; TB had higher normalized activity in one group than PM.
Keogh et. al. 1999 [10]	Six repetitions isokinetics in ecc; PM: 85 ± 14.9, TB: 77 ± 11.2* Six repetitions isokinetics in con; PM: 88 ± 18.2, TB: 80 ± 11.2* Ecc six repetitions 110% RM; PM: 85 ± 13.1, TB: 74 ± 16.7* Func isometry at 160° elbow angle; PM: 70 ± 12.7, TB: 54 ± 17.8 Func isometry at 160° elbow angle; PM: 87 ± 16.4, TB: 94 ± 17.5 Super slow 5 s ecc 55% 1 RM; PM: 57 ± 20.4, TB: 42 ± 15.2* Super slow 5 s con 55% 1 RM; PM: 82 ± 15, TB: 74 ± 16.8*; %MVIC peak, iEMG	The eccentrics and isokinetics condition had significantly greater levels of integrated EMG than heavy weight training during the eccentric phase. Likewise, functional isometrics had significantly higher TB EMG than heavy weight training in the concentric phase. Super slow motion and maximal power training both recorded significantly lower levels of force and integrated EMG than heavy weight training in each phase.
Snyder and Fry 2012 [11]	50, 80% of 1RM; PM: 92.6, 147.9, AD: 71.1, 122.9, TB: 79.8*, 124.7*; %MVIC mean RMS	During 50% lift, the verbal instruction to focus on PM or TB can increased its activity. When 80% of 1 RM is used, only the focus on the chest has been found to increase PM and AD amplitude.
Moras et. al. 2010 [13]	180°and 90°elbow flexion; MVIC values has been reported as a graph; %MVIC from peak to peak amplitude	PM, TB and AD increases its activity along with vibration frequency. The %MVIC values point out that TB activity was bigger than PM and AD activity. Furthermore, PM (%MVIC) activity was bigger than AD when vibration was applied.
Sakamoto and Sinclair 2012 [15]	40, 50, 60, 80% of 1RM at speeds of 5.6 s, 2.8 s, 1.9 s per repetition; MVIC values has been reported as a graph; Measured each 20% of the lift. RMS amplitudes and median power frequencies were normalized to two repetitions of bench press at 60% 1RM under the medium speed (2.8 s per repetition).	The main effects of fatigue, speed, and intensity were all significant for PM and TB with the amplitude being greater for the speed-failure lift, faster speeds, and heavier (higher) intensities. During the fast condition PM produce greater frequencies than the medium and slow conditions. TB decline in frequency after fatigue was greater during slower speeds. Smith Machine.
Calatayud et. al 2015 [20]	Six RM; PM mean: (mean ± SEM) 53 ± 1.9 AD mean: 60 ± 3.5 PM peak: 140 ± 6.7 AD peak: 139 ± 7.7; mean and peak %MVIC	The normalized activity of PM and AD were similar. Smith Machine.
Calatayud et. al 2016 [21]	20, 40, 50, 60, 80% of 1 RM; PM: regular BP (95% confidence interval)/PM focus/TB focus at 20% - 21 (16–25)/28 (23–32)/20 (15–24) at 40% - 38 (34–43)/44 (39–48)/40 (35–44) at 50% - 52 (47–56)/57 (53–62)/55 (51–60) at 60% - 56 (52–61)/65 (61–70)/ 61 (57–66) at 80% - 81 (77–86)/80 (75–84)/82 (77–87), TB: regular BP/PM focus/TB focus at 20% - 31 (26–36)/32 (27–36)/ 42 (37–47) at 40% - 47 (42–52)/46 (41–50)/53 (48–58) at 50% - 55 (50–60)/54 (49–59)/59 (54–64) at 60% - 60 (55–65)/59 (54–64)/64 (60–69) at 80% - 80 (75–85)/81 (76–85)/82 (78–87); %MVIC in peak RMS (average of 3 reps)	In both muscles, focusing on using the respective muscles increased muscle activity at relative loads between 20 and 60% but not at 80% of 1RM. Both muscles show similar activity.
Schoenfeld et. al 2016 [22]	80, 50% of 1RM mean/peak; PM S: 121 ± 33, 103 ± 39/308 ± 121, 305 ± 179, PM C: 127 ± 45, 117 ± 53/321 ± 121, 329 ± 167, AD: 115 ± 39, 105 ± 44/275 ± 102, 272 ± 128, TB: 94 ± 30, 69 ± 23/237 ± 109, 202 ± 91; %MVIC, mean, peak, and iEMG muscle activation	The PM showed higher MVIC values than TB and AD.

ecc = eccentric, con = concentric, EMG = electromyography,

*highest value reported at condition,

iEMG = integrated electromyography, values are the mean ± standard deviation if not specified other, %MVIC = percentage of maximum voluntary isometric contraction, AD = deltoid anterior, PM = pectoralis major, TB = triceps brachii, SEM = standard error of measurement, RMS = route mean square, RM = repetition maximum, S = sternal portion of PM, C = clavicular portion of PM, Func = functional.

Selected studies had different research foci. This allowed the identification of different conditions that can cause changes in muscle activity. It has been found that changes in muscle activity are caused by exercise intensity [10, 11, 17, 21, 22], velocity of movement [15], fatigue [15], mental focus [11, 21], movement phases [18] and stability conditions [13, 19], such as bar vibration or unstable surfaces. The TB is most often the object of activity change under such conditions (Tables 2 and 3).

**Table 3:** Summary of the effects of bench press exercise conditions on muscle activity.

Parameter	Effect
**BP exercise Intensity (load)**	Increase in intensity is resulting in increased amplitude of PM [10, 11, 17, 21, 22], TB [11, 22], and AD [11, 17, 22].
**Velocity of movement**	EMG amplitude increases with increased speed of movement in PM, TB, [15].PM EMG frequency increases with increased speed [15].TB decline in EMG frequency after fatigue during slower speeds [15].
**Stability condition**	The PM, TB and AD increases its activity along with bar vibration frequency [13].RMS values increases with increasing instability in latissimus dorsi, erector spinae, biceps femoris, soleus, internal obliques, but not in rectus abdominis [19].
**Fatigue**	EMG amplitude increases in fatigue in PM, TB, [15].EMG median frequencies before fatigue is similar among speeds and intensities and decreases after fatigue in PM, TB, AD [15].TB decline in EMG frequency after fatigue during slower speeds [15].
**Mental focus**	Focus on PM or TB during 50% of 1RM BP can increases the activity of PM and TB [11, 21].Focus on PM during intensity of 80% of 1RM can increases the activity of PM and AD [11].Focusing on TB or PM (both parts) increase their EMG amplitude at relative loads between 20 and 60%, but not at 80% of 1RM [21].
**Movement phase**	The biceps activity was higher in the pre-sticking region compared with the other regions and the TB activity increases continuously from region to region in both conditions. TB and PM increases during sticking region [18].
**Intervention**	A 12-week intervention performing, two times a week, a regular BP in 4 sets or variable ROM BP in 5 sets resulted in no EMG change [9].
**Compression sleeves**	No positive performance effects or EMG change when wearing graduated compression sleeves during power exercise in young trained men [12].

ROM = range of motion, EMG = electromyography, RT = resistance training, RM = repetition maximum, min = minimum, PM = pectoralis major, TB = triceps brachii, AD = anterior deltoid, BP = bench press,

## Discussion

Numerous differences were found in the objective of EMG studies included but not in the study design because most of the included studies used cross sectional measurements and only one applied intervention (Table 1). The diversity in research objectives resulted in variability of observed participants, although all studies referred to RT men but represented different sport disciplines and different performance levels. The included studies were similar in the number of participants (11–22) and their ages (21–35), which means that our outcomes should be interpreted specifically for male subjects of this age group. Although there were differences in the study outcomes, their results provided a sufficient amount of normalized data to answer the first research question and six different conditions that cause changes in muscle activity during the BP.

Full-text screening resulted in a high number (66) of studies rejected by specific methodological quality analyses (Fig 1), which was due to the use of surface EMG and not fine-wire electrode EMG in screened studies. Therefore, a detailed discussion about specific EMG methodological quality analyses is considered further because of its lack in the current research. Reviewers concluded that the muscle model (pattern) is not fully established across included articles (Table 1); therefore, an additional section discussing the muscle model for BP has been included. Two included articles focused on comparing BP to another exercise. Because this issue is of great practical significance, it was discussed over the primary aim of this review.

The finding that the TB and PM have higher activity than AD during the BP is reasonable in terms of their size and ability of force production in this movement. More important is the fact that TB is most often the subject of activity change during different conditions of the BP. Therefore, conditions such as exercise intensity, mental focus and movement phase have been discussed separately. Special attention should be directed to stability conditions, which differ significantly in the observed muscle model. The effect of fatigue and velocity has been fully resolved by the results of one original author (extracted in Table 3), where the PM EMG frequency increases with exercise speed and the TB frequency decreases after fatigue at slower speed [15]. The PM, TB and AD EMG median frequency decreases with increased fatigue [15], whereas the PM and TB amplitude increases as the speed increases [15]. The activity of the PM and TB can also change in response to a combination of the speed of contraction and type of muscle action (Table 2) [10].

The only intervention study [9] reported no EMG differences associated with a training protocol; therefore, an activity change should not be expected in the healthy participants during BP intervention. The use of graduated compression sleeves during the BP exercise resulted in no change in EMG amplitude [12] at 50% of 1RM; therefore, this issue does not have to be developed in future research.

### Surface EMG limitations

The most appropriate methods to gather and evaluate surface EMG can be debated, especially during dynamic movements. This occurs because surface electrodes shift toward the innervation zone during dynamic movements, which means that any changes in the muscle length or joint angles would affect the resultant surface EMG signals [7]. Therefore, the surface EMG signal must be measured at a known joint angle or at least a known speed of movement, which was reflected in the full text exclusion criteria. Of the studies included in the current review, only one study reported the joint angles in conjunction with EMG during dynamic BP [17]; however, some studies graphically reported the EMG in relation to a percentage of movement phase [30–33], but they did not provide angle-specific data. One study reported angle-specific muscle activity in an isometric condition with vibration [13]. Five studies [9, 10, 13, 17, 34] used isometric muscle action to gather the EMG, and 9 studies included a pre-defined control of the speed of the BP task (Table 1). There were also some studies that pre-defined the speed of the movement but were focused on comparison of EMG amplitude between different joint angles, such as different bench inclinations [35–37], which would be contradictory to the first quality inclusion criteria. In contrast, the included studies differed slightly in their instructions for grip width (e.g., wider than shoulder width or grip wide derived from 90° elbow flexion position during the BP). However, those studies did not vary in trunk inclination, and the grip width differences were negligible compared to the changes in inclination.

Additionally, because many studies do not use congruous terminology (i.e., activation, EMG activity, and amplitude), statements that consider amplitude to be a direct measure of muscle activity have been used interchangeably in the discussion. For future BP studies, the EMG recommendations should include general guidelines and should extensively report joint angle, movement speed, muscle fatigue, exercise intensity, and appropriate normalization methods. Furthermore, the signal analyses should acknowledge that muscle unit recruitment depends on the load and should be analyzed by spike trigger averaging or initial wavelet analyses followed by tuning wavelets to major EMG frequencies [4]. Therefore, a large number of studies were rejected, although they have historically proven to have merit within the field. It is important to note that this paper is not an empirical one, but it is a review of the up-to-date studies related to a well-controlled EMG analysis of the BP movement.

This review also does not refer to any data obtained by fine-wire electrodes; however, no studies matching the selection and inclusion criteria used the fine-wire electrode technique or high-density surface EMG. Therefore, the use of fine-wire electrodes and high-density surface EMG is recommended for future studies. Due to the variability between methodological approaches in particular studies, the global interpretation of EMG data should be interpreted with caution.

### Muscle models

Each EMG study must be based on a “muscle model” that is selected for a specific exercise. The traditional muscle model for the BP was based on prediction by biomechanical lever arms and empirical knowledge, where the pectoralis major (PM), anterior deltoid (AD) and triceps brachii (TB) were estimated as the primary movers. This specific muscle model was first applied by Elliott [38], where the sternal portion of PM and the long head of TB were used. Later, Barnet [37] suggested that the latissimus dorsi (LD) may play an important role as a primary mover. His suggestion has also been considered in other studies [16, 19].

The PM has two separate innervations (by medial and lateral pectoral nerves) in the clavicular and sternal portion, where the measurement of the sternal portion seems to be appropriate for the measurement of PM activity as a primary mover during the flat BP. If a comparison of the BP to its variability or a different exercise is considered, then the measurement of the sternal and clavicular portions seems justified [16].

The TB has three anatomical heads (subdivisions). None of the BP studies justified the choice of measuring particular heads of the TB; moreover, four studies did not report an exact subdivision for electrode placement (Table 1). This may be confusing because the lateral and medial TB head is innervated by the radial nerve and the long head is innervated by the axillary nerve [39]. Only one study simultaneously measured all three heads of the triceps [21]. Because there is no comparison of the activity of particular TB subdivisions, it is difficult to determine which subdivision is appropriate for a specific measurement.

The AD measurement has been established as a standard, where the deltoid is divided into three anatomical subdivisions (anterior, middle and posterior part). None of the studies have considered the fact that the AD can be divided into seven subdivisions defined by intramuscular tendons [40]. Those seven divisions can be independently coordinated by the central nervous system [41] and may be useful for a detailed comparison of AD activity in BP variations. The medial portion of the deltoid has not been measured in any study (Table 1). The posterior deltoid has been measured for its antagonist function to AD when the arm is abducted to nearly 90° [11, 16].

Neuromuscular activity is not only dependent on the co-activation of primary movers but also on the activation of their antagonist muscles as stabilizers. This was considered for the biceps brachii muscle and posterior deltoid, as the antagonist for TB and AD, respectively [11, 19, 34]. Muscle activity, when evaluated under different stability conditions or in comparison to other exercises similar to the BP, enlarged the muscle model to abdominal muscles, erector spinae muscles and several others, as presented in Table 1. These muscle models varied due to the type of compared exercise or instability condition [19].

With the principal question of measuring the muscles responsible for force output, the long head of TB, lateral or medial head of TB, sternal portion of pectoralis major, anterior deltoid, medial deltoid and latissimus dorsi should be measured. However, the exact role of each TB head during the BP should be further investigated, as should the medial deltoid or more detailed subdivisions of deltoid muscles. The biceps brachii acts as a stabilizer and antagonist, which is the principal reason to include its measurement. Other antagonist or stabilizer muscles are useful if there is a specific justification for their use.

### Comparison of BP with other exercises

Strength training protocols are typically composed from sets of exercises, where the muscle involvement plays an important role in exercise selection. In some cases, the training objective is to target the same muscle groups, and in others, the objective is to not overlap the involvement of trained muscles. For example, during the pec deck exercise (seated humerus adduction with the arms and elbows flexed at 90°), the PM and AD muscle activities have been found to be higher than TB, whereas the TB activity was found to be higher for the BP than the pec deck exercise. Another study [16] reported a higher EMG amplitude of the PM and AD muscles in the BP compared with the barbell pullover. In reverse order, the activity of LD and the lateral head of the TB was higher in the pullover than in the BP [16]. Therefore, the pec deck, pullover and BP could be performed with the purpose of preferentially stimulating the AD and PM muscles or the TB, depending on the training needs [14, 16]. In contrast, the 6RM bench press and 6RM elastic band push-up resulted in similar muscle activity [20], where the EMG amplitude during the push-up was 52.9 ± 2.55% MVIC in PM and 62.32 ± 2.87% MVIC in AD, whereas the BP resulted in values of 52.7 ± 1.85% MVIC in PM and 59.53 ± 3.54% MVIC in AD.

### Muscle activity change along with exercise intensity

A change in muscle activity along with exercise intensity requires a specific approach to analyze the exercise protocol and data acquisition. Raw data were often expressed as RMS in Volts, integrated EMG or RMS normalized to maximal voluntary isometric contraction (MVIC), while only two studies (Table 2) used frequency analyses [11, 15], which is preferentially recommended for estimating muscle activity along with changes in exercise intensity. The finding that exercise intensity resulted in increased amplitude (Table 3) may seem obvious. However, the finding that exercise intensity interacts with other conditions such as speed, fatigue [15] and mental focus [11] suggest its key role in determining the activity level. The activity level of TB, PM and AD typically exceed the 100% of MVIC when the exercise intensity is above 80% of 1 RM and peak amplitude is observed (Table 2). This seems to be reasonable because dynamic movement with heavy loads typically produces more interactive force than isometric conditions.

One of the innovative ways to increase exercise intensity includes vibration training where 45 Hz elicited a higher EMG amplitude than 50 Hz did in elbow vibration. However, the elbow flexion vibration should be higher than 25 Hz to induce increases in EMG amplitude in the deltoid and PM [13]. The studies evaluating EMG amplitude change along with increased load are summarized in Table 2 with their major outcomes, but studies evaluating EMG frequency changes are scarce.

### Mental focus

The voluntary focus or verbal technique instruction can modify muscle activity, although it is dependent on exercise experience [42] and exercise intensity [21]. In experienced athletes, placing an intentional focus on the arms and chest has been found to increase the amplitude of these regions during the BP (Tables 2 and 3) at relative loads between 20 and 60% but not at 80% of 1RM [11, 21]. When 80% of 1 RM is used, only the focus on the chest has been found to increase the PM and AD amplitude [11]. Although mentally focusing on specific regions impacts the acute muscle activity in those regions, it is unclear whether greater EMG amplitudes are indeed associated with greater hypertrophy, strength, or improvements in functional motor tasks [43].

### Stable and unstable surfaces

Unstable surface training became a popular method to increase exercise variability and stability. Unfortunately, this method is often used for the aims that are not appropriate for its effective use. It was suggested that during BP, an unstable surface does not produce a higher EMG amplitude of primary movers than a stable surface [44] in 1RM, and a stable surface has been found to have greater TB and PM activity compared with unstable surfaces in 6RM BP [45]. In contrast, if the objective is to target body stabilizers, an unstable environment has been found to effectively increase the activation of the trunk stabilizing musculature [19].

### Movement phase—Sticking region

The 1RM in the BP is typically performed with a partial decrease of lifting speed (deceleration) called the sticking region. The first EMG evaluation of sticking regions was performed by Elliott [38], who suggested that the appearance of the sticking region is not the cause of decreased muscular activity [38]. A recent study using 12 isometric positions of muscle activity confirmed that the sticking region is the result of a poor mechanical force position rather than a lack of muscle excitation in the prime movers, which cannot explain the existence of the sticking region [18]. The sticking period occurs in both successful and unsuccessful attempts in maximal bench press performance, but the failure does not always occur during the sticking region [46]. During the BP, the biceps brachii muscle has been found to decrease activity from the pre-sticking region to the sticking region. The TB activity increased from the eccentric phase to the pre-sticking region and from the pre-sticking region to the sticking and the post-sticking region, with no differences in muscle amplitude between the sticking and post-sticking regions [47]. The TB and PM activity increases during the sticking region [18], which suggests that those two muscles are responsible for surpassing this point. The occurrence of the sticking region is not only the domain in the flat BP, but it also exists in the dumbbell chest press [47] and squat [48].

## Conclusions

Exercise intensity is the key factor that can change muscle activity, where the intensity interacts with other exercise conditions during the BP. Furthermore, the effects of velocity of movement, fatigue, mental focus, movement phases and stability conditions have been demonstrated, but large and unresolved areas of research remain. The PM and TB have higher EMG amplitudes and more dominant roles than the AD during the BP, where the TB EMG is most often the subject of change. However, the muscle model of prime movers during the BP is not sufficiently established in terms of which muscle subdivisions should be measured as a standard procedure or during special circumstances such as unstable surfaces. Future studies on bench press performance should investigate the poor mechanical force production in the sticking region by means of leverage changes. The following major topics should be investigated in the future:

1. Developing a clear musculoskeletal model of BP primary movers, including all subdivisions and latissimus dorsi.
2. Establishing clear functions and definitions of BP muscle stabilizers.
3. Determining muscles with the greatest potential to overcome sticking regions in the BP.
4. Evaluation of muscular activity during the BP movement with a varied time of contraction.
5. Determining changes in muscle activity and performance following different training protocols.
6. Establishing the changes in EMG activity and kinematic variables in numerous repetitions of the BP while developing strength endurance.
7. Establishing innovative methods for evaluating EMG during dynamic BP movements.
8. Comparison of surface electrode measurements during the BP to the fine-wire electrodes measurement and high-density surface EMG.

## Supporting information

S1 Table The modified STROBE checklist, Von Elm et al., 2007 [9]. 9. Von Elm E, Altman DG, Egger M, Pocock SJ, Gøtzsche PC, Vandenbroucke JP. The Strengthening the Reporting of Observational Studies in Epidemiology (STROBE) Statement: Guidelines for reporting observational studies. Prev Med. 2007;45(4):247–51. doi: http://dx.doi.org/10.1016/j.ypmed.2007.08.012.
(DOCX)

S2 Table Strengthening the Reporting of Observational Studies in Epidemiology (STROBE) checklist. (DOCX)

S1 Protocol The review protocol and data extraction form used during the review process. (PDF)
