# Supplementary material for: A systematic review of surface electromyography analyses of the bench press movement task
Source: PLoS One. 2017 Feb 7;12(2):e0171632. doi: 10.1371/journal.pone.0171632 (PMC5295722; doi:10.1371/journal.pone.0171632)
Supplement: S1 Table — 9. Von Elm E, Altman DG, Egger M, Pocock SJ, Gøtzsche PC, Vandenbroucke JP. The Strengthening the Reporting of Observational Studies in Epidemiology (STROBE) Statement: Guidelines for reporting observational studies. Prev Med. 2007;45(4):247–51. doi: http://dx.doi.org/10.1016/j.ypmed.2007.08.012. (DOCX) [file pone.0171632.s001.docx]

**S1 Table. The modified STROBE checklist, Von Elm et al., 2007 [**[**1**](#_ENREF_1)**].**

|  | n | Description |
| --- | --- | --- |
| TITLE /  ABSTRACT | 1 | Indicate the study's design with a commonly used term in the title or the abstract. Provide in the abstract an informative and balanced summary of what was done and what was found including bench press measurement. |
| INTRODUCTION | 2 | Explain the scientific background and rationale for the investigation being reported in introduction. |
|  | 3 | State specific objectives, including any pre-specified hypotheses in introduction. |
| METHODS | 4 | Present key elements of study design early in the paper such as in “Methods”. |
|  | 5 | Describe the setting, locations, and relevant dates, including periods of recruitment, follow-up, and data collection. Describe methods of follow-up. |
| *Participants* | 6 | Participant eligibility criteria, and the sources and methods of selection of participants. Give matching criteria of participants, strength training experience. |
| *Test methods* | 7 | Clearly define all outcomes, exposures, predictors, potential confounders, and effect modifiers. Give diagnostic criteria, if applicable. |
|  | 8 | For each variable of interest, give sources of data and details of methods of assessment (measurement). Describe comparability of assessment methods if there is more than one group. |
|  | 9 | Describe any efforts to address potential sources of bias. |
|  | 10 | Explain how the study size was arrived at. |
|  | 11 | Explain how EMG raw data were acquisitioned and handled in the analyses. If applicable, describe which groupings were chosen, and why. |
|  | 12 | Describe all statistical methods, including those used to control for confounding. Describe any methods used to examine subgroups and interactions. If applicable, describe analytical methods taking account of sampling strategy. Describe any sensitivity analyses. |
| RESULTS | 13 | Report the numbers of individuals at each stage of the study, completing follow-up, and analysed in results. Indicate the number of participants with missing data for each variable of interest. Explain how missing data were addressed. |
|  | 14 | Report numbers of outcome events or summary measures. |
| *Test results* | 15 | Give unadjusted estimates of EMG outcome and, if applicable, their precision (e.g., 95% confidence intervals). Make clear which confounders were adjusted for and why they were included in Main results. |
|  | 16 | Report other analyses done—e.g., analyses of subgroups and interactions, and sensitivity analyses.  Other analyses. |
| *Discussion* | 17 | Summarise key results with reference to study objectives in discussion. |
|  | 18 | Discuss limitations of the study, taking into account sources of potential bias or imprecision. Discuss both direction and magnitude of any potential bias. |
|  | 19 | Give a cautious overall interpretation of results considering objectives, limitations, multiplicity of analyses, results from similar studies, and other relevant evidence. |
|  | 20 | Discuss the generalisability (external validity) of the study results. |

1. Von Elm E, Altman DG, Egger M, Pocock SJ, Gøtzsche PC, Vandenbroucke JP. The Strengthening the Reporting of Observational Studies in Epidemiology (STROBE) Statement: Guidelines for reporting observational studies. Prev Med. 2007;45(4):247-51. doi: <http://dx.doi.org/10.1016/j.ypmed.2007.08.012>.
