# Supplementary material for: A systematic review of surface electromyography analyses of the bench press movement task
Source: PLoS One. 2017 Feb 7;12(2):e0171632. doi: 10.1371/journal.pone.0171632 (PMC5295722; doi:10.1371/journal.pone.0171632)
Supplement: S2 Table — (DOCX) [file pone.0171632.s002.docx]

**S2 Table. Strengthening the Reporting of Observational Studies in Epidemiology (STROBE) checklist**

| **Item** | **1** | **2** | **3** | **4** | **5** | **6** | **7** | **8** | **9** | **10** | **11** | **12** | **13** | **14** | **15** | **16** | **17** | **18** | **19** | **20** |
| --- | --- | --- | --- | --- | --- | --- | --- | --- | --- | --- | --- | --- | --- | --- | --- | --- | --- | --- | --- | --- |
| **(**[**Martorelli, Martorelli et al. 2014**](#_ENREF_6)**)** | **Y** | **Y** | **Y** | **Y** | **N** | **Y** | **Y** | **N** | **N** | **N** | **Y** | **Y** | **N** | **Y** | **Y** | **Y** | **Y** | **N** | **Y** | **Y** |
| **(**[**Campos and Da Silva 2014**](#_ENREF_3)**)** | **Y** | **Y** | **Y** | **Y** | **N** | **Y** | **Y** | **N** | **N** | **N** | **Y** | **Y** | **N** | **Y** | **Y** | **N** | **Y** | **N** | **Y** | **Y** |
| **(**[**Clark, Humphries et al. 2011**](#_ENREF_4)**)** | **Y** | **Y** | **Y** | **Y** | **N** | **Y** | **Y** | **Y** | **N** | **N** | **Y** | **Y** | **N** | **Y** | **Y** | **Y** | **Y** | **Y** | **Y** | **Y** |
| **(**[**Keogh, Wilson et al. 1999**](#_ENREF_5)**)** | **Y** | **Y** | **Y** | **Y** | **N** | **Y** | **Y** | **N** | **N** | **N** | **Y** | **Y** | **N** | **Y** | **Y** | **Y** | **Y** | **N** | **Y** | **Y** |
| **(**[**Moras, Rodriguez-Jimenez et al. 2010**](#_ENREF_7)**)** | **Y** | **Y** | **Y** | **Y** | **N** | **N** | **Y** | **N** | **N** | **N** | **Y** | **Y** | **N** | **Y** | **Y** | **Y** | **Y** | **N** | **Y** | **Y** |
| **(**[**Ojasto and Häkkinen 2009**](#_ENREF_9)**)** | **Y** | **Y** | **Y** | **Y** | **N** | **Y** | **Y** | **N** | **N** | **N** | **Y** | **Y** | **N** | **Y** | **N** | **Y** | **Y** | **N** | **Y** | **Y** |
| **(**[**Norwood, Anderson et al. 2007**](#_ENREF_8)**)** | **Y** | **Y** | **Y** | **Y** | **N** | **Y** | **Y** | **N** | **N** | **N** | **Y** | **Y** | **N** | **Y** | **N** | **Y** | **Y** | **N** | **Y** | **Y** |
| **(**[**Tillaar, Saeterbakken et al. 2012**](#_ENREF_14)**)** | **Y** | **Y** | **Y** | **Y** | **N** | **Y** | **Y** | **N** | **N** | **N** | **Y** | **Y** | **N** | **Y** | **N** | **Y** | **Y** | **Y** | **Y** | **Y** |
| **(**[**Sakamoto and Sinclair 2012**](#_ENREF_11)**)** | **Y** | **Y** | **Y** | **Y** | **N** | **Y** | **Y** | **N** | **N** | **N** | **Y** | **Y** | **N** | **Y** | **N** | **Y** | **Y** | **N** | **Y** | **Y** |
| **(**[**Rocha Jr, Gentil et al. 2007**](#_ENREF_10)**)** | **Y** | **Y** | **Y** | **Y** | **N** | **Y** | **Y** | **N** | **N** | **N** | **Y** | **Y** | **N** | **Y** | **N** | **Y** | **Y** | **N** | **Y** | **Y** |
| **(**[**Schoenfeld, Contreras et al. 2016**](#_ENREF_12)**)** | **Y** | **Y** | **Y** | **Y** | **N** | **Y** | **Y** | **N** | **N** | **N** | **Y** | **Y** | **N** | **Y** | **Y** | **Y** | **Y** | **N** | **Y** | **Y** |
| **(**[**Snyder and Fry 2012**](#_ENREF_13)**)** | **Y** | **Y** | **Y** | **Y** | **N** | **Y** | **Y** | **N** | **N** | **N** | **Y** | **Y** | **N** | **Y** | **N** | **Y** | **Y** | **N** | **Y** | **Y** |
| **(**[**Calatayud, Borreani et al. 2015**](#_ENREF_1)**)** | **Y** | **Y** | **Y** | **Y** | **N** | **Y** | **Y** | **N** | **N** | **N** | **Y** | **Y** | **N** | **Y** | **N** | **Y** | **Y** | **N** | **Y** | **Y** |
| **(**[**Calatayud, Vinstrup et al. 2016**](#_ENREF_2)**)** | **Y** | **Y** | **Y** | **Y** | **N** | **Y** | **Y** | **N** | **N** | **N** | **Y** | **Y** | **N** | **Y** | **N** | **Y** | **Y** | **N** | **Y** | **Y** |

**References**

Calatayud, J., S. Borreani, J. C. Colado, F. Martin, V. Tella and L. L. Andersen (2015). "Bench Press and Push-up at Comparable Levels of Muscle Activity Results in Similar Strength Gains." J Strength Cond Res **29**(1): 246-253.

Calatayud, J., J. Vinstrup, M. D. Jakobsen, E. Sundstrup, M. Brandt, K. Jay, J. C. Colado and L. L. Andersen (2016). "Importance of mind-muscle connection during progressive resistance training." European Journal of Applied Physiology **116**(3): 527-533.

Campos, Y. D. A. C. and S. F. Da Silva (2014). "Comparison of electromyographic activity during the bench press and barbell pullover exercises." Motriz. Revista de Educacao Fisica **20**(2): 200-205.

Clark, R. A., B. Humphries, E. Hohmann and A. L. Bryant (2011). "The influence of variable range of motion training on neuromuscular performance and control of external loads." Journal of Strength and Conditioning Research **25**(3): 704-711.

Keogh, J. W. L., G. J. Wilson and R. P. Weatherby (1999). "A Cross-Sectional Comparison of Different Resistance Training Techniques in the Bench Press." Journal of Strength and Conditioning Research **13**(3): 247-258.

Martorelli, S. S., A. S. Martorelli, M. C. Pereira, V. A. Rocha-Junior, J. G. Tan, J. G. Alvarenga, L. E. Brown and M. Bottaro (2014). "Graduated compression sleeves: effects on metabolic removal and neuromuscular performance." J Strength Cond Res **29**(5): 1273-1278.

Moras, G., S. Rodriguez-Jimenez, J. Tous-Fajardo, D. Ranz and I. Mujika (2010). "A vibratory bar for upper body: feasibility and acute effects on EMGrms activity." J Strength Cond Res **24**(8): 2132-2142.

Norwood, J. T., G. S. Anderson, M. B. Gaetz and P. W. Twist (2007). "Electromyographic activity of the trunk stabilizers during stable and unstable bench press." Journal of Strength and Conditioning Research **21**(2): 343-347.

Ojasto, T. and K. Häkkinen (2009). "Effects of different accentuated eccentric loads on acute neuromuscular,growth hormone, and blood lactate responses during a hypertrophic protocol." Journal of Strength and Conditioning Research **23**(3): 946-953.

Rocha Jr, V. D. A., P. Gentil, E. Oliveira and J. Do Carmo (2007). "Comparison among the EMG activity of the pectoralis major, anterior deltoidis and triceps brachii during the bench press and peck deck exercises." Revista Brasileira de Medicina do Esporte **13**(1): 43e-46e.

Sakamoto, A. and P. J. Sinclair (2012). "Muscle activations under varying lifting speeds and intensities during bench press." Eur J Appl Physiol **112**(3): 1015-1025.

Schoenfeld, B. J., B. Contreras, A. D. Vigotsky, D. Ogborn, F. Fontana and G. Tiryaki-Sonmez (2016). "Upper body muscle activation during low-versus high-load resistance exercise in the bench press." Isokinetics and Exercise Science(Preprint): 1-8.

Snyder, B. J. and W. R. Fry (2012). "Effect of verbal instruction on muscle activity during the bench press exercise." Journal of Strength and Conditioning Research **26**(9): 2394-2400.

Tillaar, R., A. H. Saeterbakken and G. Ettema (2012). "Is the occurrence of the sticking region the result of diminishing potentiation in bench press?" J Sports Sci **30**(6): 591-599.
