# Supplementary material for: A systematic review of surface electromyography analyses of the bench press movement task
Source: PLoS One. 2017 Feb 7;12(2):e0171632. doi: 10.1371/journal.pone.0171632 (PMC5295722; doi:10.1371/journal.pone.0171632)
Supplement: S1 Protocol — (PDF) [file pone.0171632.s003.pdf]

Review title

A Systematic Review of Muscle Activity during the Bench Press Exercise

Reviewers

Petr Stastny, Charels University in Prague, Faculty of Physical Education and Sport
Artur GoğaŜ, The Jerzy Kukuczka Academy of Physical Education in Katowice
Dusan Blazek, Charels University in Prague, Faculty of Physical Education and Sport
Adam Maszczyk, The Jerzy Kukuczka Academy of Physical Education in Katowice
MichağWilk, The Jerzy Kukuczka Academy of Physical Education in Katowice
Petr Uhlir, Palacky University in Olomouc, Faculty of Physical Culture
Przemysğaw Pietraszewski, The Jerzy Kukuczka Academy of Physical Education in Katowice
Miroslav Petr, Charels University in Prague, Faculty of Physical Education and Sport
Adam ZajŃc, The Jerzy Kukuczka Academy of Physical Education in Katowice
Provedene kroky: Abstract and title screening (PS, AG, DB)

Center conducting the review

Charles University in Prague, Faculty of Physical Education and Sport, Library

Review question/objective

What have previous electromyography studies described in the bench press (BP) exercise?

The activity of which muscles has been studied?

Which muscles show the greatest activity during the BP?

What are the future directions of EMG research regarding the BP?

What can alternate the muscle activity during BP?

More specifically, the objectives are to identify:

The objective of this paper is to review well controlled electromyography (EMG) and kinematic studies performed on the barbell BP exercise.

Background (short)

Scientists, coaches and athletes are interested in details related to maximum strength, explosive strength improvement or power output during the BP exercise as well as muscle activity between BP variations. Previous reviews have been written to evaluate the criteria for BP efficiency and safety that can be prescribed in conditioning programs and how to set up optimal load for power training. However, muscle activity has been largely studied without summarizing current findings.

Created by XMLmind XSL-FO Converter.

Inclusion criteria

Types of participants

The quantitative component of review will consider studies that include any number on participants, important is the qualitative parameter.

The qualitative component of review will consider studies that include resistance trained participants, where resistance trained can be determined as following: the resistance training experience for more than 6month, performing sport discipline where the resistance training is basic part of conditioning, lifting at least own body weight for BP, no injury during and prior to the measurement, no recondition or other health related problems.

Example: statement that ñresistance trainedòparticipant has been included; rugby league players with a minimum 6 months of resistance training experience, Resistance trained student able to lift at lest their body weight on bench press.

Interest: Any.

Types of outcomes

Any objective electromyography measurements on BP exercise.

Review will consider studies that include the following outcome measures: root mean square EMG values during BP, mean and peak EMG values during BP, any normalized EMG amplitude values during BP, integrated EMG, EMG frequency analyses, the muscle activity onset during BP.

Exclusion criteria: full text was not available in English, the study did not contain an appropriate description of measuring devices and procedures, the study did not include a proper exercise task and the study did not report how raw EMG data were processed.

Types of studies

The review will consider studies that evaluate electromyography (EMG) in cohort study, analytical cross sectional study, randomized control trials, non-randomized control trials, interventional study, case control studies and others which includes at least one time measurement of EMG during BP.

The review studies will not be accepted as included ones, but it would be used for hand search in their reference list.

The dissertation thesis, conference proceedings, conference monography and other reviews would not be accepted. The retrospective studies would not be accepted because the area of interest belong to data which requires to perform experiment.

The qualitative component of the review will consider the use of BP protocol and justification for processing the EMG data, but not limited to, reviewer will consider also general methodological designs. The textual component of the review will consider expert opinion, discussion papers, position papers and other text.

Created by XMLmind XSL-FO Converter.

Search strategy

A systematic computerized literature search will be conducted in PubMed (1940 to search date), Scopus (1823 to search date), Web of Science (1974 to search date) and the Cochrane Central Register of Controlled Trials (CENTRAL) in the Cochrane Library.

A search formula should include the synonyms for bench press exercise, measurement and electromyography. The search formula will be finalized after reviewer discussion. Only original, full text articles would be qualified and not Comment, Proceedings, Editorial or Letter. The search did not include conference abstracts and dissertations. The reviews will be used for hand search in their reference list. In addition, a hand search will done on the reference list of included articles.

Formula: (bench press) OR (chest press) OR (board press) AND (test OR measure* OR assessment OR dynamometer OR kinematics OR biomechanics) NOT (Comment [pt] OR Proceeding OR Editorial [pt] OR Letter[pt]).

Assessment of methodological quality

General methodology:

By adopted STROBE checklist which includes also bias assessment. Not rejected if any part is missing.

Specific methodology:

The detailed description of EMG measurement, which ensures the control of BP movement and EMG signal. The use of any kinds of electrodes.

Discussion on further inclusion will be necessary.

Data collection

By extraction form, as much as possible.

Data synthesis

The use of meta-analyses will be decided after data collection. Data would be compared based on reviewers expertise.

Acknowledgements

This work was supported by the Grant Agency of Czech Republic under Grant NO. 16-13750S, PROK P38 grant and Ministry of Science and Higher Education of Poland under Grant NRSA3 03953 and NRSA4 040 54.

Created by XMLmind XSL-FO Converter.

Data extraction form

Reviewer.. Date.....................................

Author.. Journal...............................

Study method

Cross sectional Intervention RCT

Other:

Study aim:____

____

Participants

n =.................settings (age, height, weight,other):

population:

Resistance training experience description:

EMG data

Bench press description:...

Movement speed control:...

EMG raw data procesing:...

EMG units used for results:..

EMG values/condition:

EMG normalisation method:...

Authors conclusions:

____

____

____

____

Reviewer decision:

____

____

____

Created by XMLmind XSL-FO Converter.
